# DHA/AA alleviates LPS-induced Kupffer cells pyroptosis via GPR120 interaction with NLRP3 to inhibit inflammasome complexes assembly

**DOI:** 10.1038/s41419-020-03347-3

**Published:** 2021-01-12

**Authors:** Guoqiang Fan, Yanfei Li, Jinglong Chen, Yibo Zong, Xiaojing Yang

**Affiliations:** MOE Joint International Research Laboratory of Animal Health and Food Safety, Nanjing Agricultural University, Nanjing, 210095 P. R. China

**Keywords:** Cell death, Immunology

## Abstract

Pyroptosis is a novel type of programmed cell death associated with the pathogenesis of many inflammatory diseases. Docosahexaenoic acid (DHA) and Arachidonic acid (AA) is widely involved in inflammatory pathological processes. However, the effect and mechanism of DHA and AA on pyroptosis in Kupffer cells are poorly understood. The present study demonstrated that DHA and AA ameliorated lipopolysaccharide (LPS)-induced Kupffer cells pyroptosis by reversing the increased expression of NLRP3 inflammasome complex, GSDMD, IL-1β, IL-18, and PI-stained positive rate. Next, the study revealed that GPR120 silencing eliminated the anti-pyroptosis of DHA and AA in LPS-induced Kupffer cells, suggesting that DHA and AA exerted their effect through GPR120 signaling. Importantly, GPR120 endocytose and binds to NLRP3 under LPS stimulation. Furthermore, co-immunoprecipitation showed that DHA and AA promoted the interaction between GPR120 and NLRP3 in LPS-exposed Kupffer cells, thus inhibiting the self-assembly of NLRP3 inflammasome complex. Finally, the study verified that DHA and AA alleviated hepatic injury through inhibiting Kupffer cells pyroptosis in vivo. The findings indicated that DHA and AA alleviated LPS-induced Kupffer cells pyroptosis via GPR120 interaction with NLRP3, it might become a potential therapeutic approach hepatic injury.

## Abstract (web summary)

DHA/AA alleviated LPS-induced Kupffer cells pyroptosis via GPR120 interaction with NLRP3 to inhibit NLRP3 inflammasome assembly.

## Graphical abstract

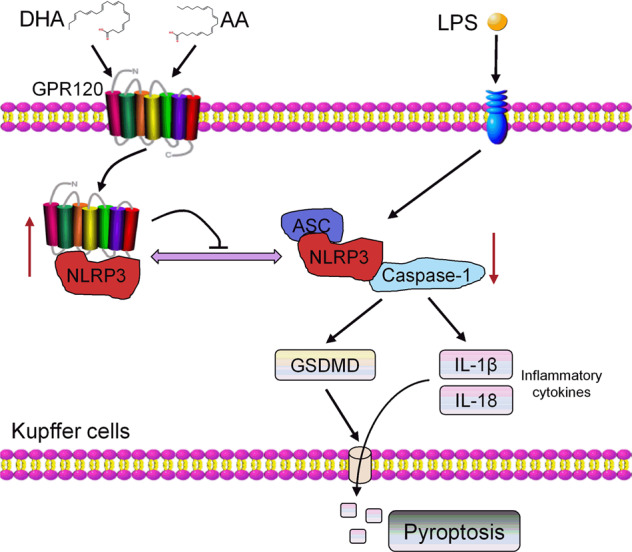

## Introduction

The liver is the largest organ in the body responsible for nutrients metabolism. Hepatic injury is perceived as one of the major problems threatening human health; it is often associated with increased hepatic exposure to lipopolysaccharide (LPS). In the microenvironment of the liver, Kupffer cells are the first barrier defending against pathogens by sensing damage stimulus and producing various cytokines^1,2^. The innate-immune-dominated tissue inflammatory response initiated by Kupffer cells after hepatic injury involves many regulatory mechanisms^3,4^.

Pyroptosis is a novel type of programmed cell death discovered and proved in recent years. It is initiated by the activation of NOD-like receptor pyrin domain-containing protein 3 (NLRP3) inflammasome and performed by gasdermin D (GSDMD) protein. As a result, the activated GSDMD constitutes nanopores in the cell membrane, resulting in cell swelling, cell membrane rupture, and even cell death^5,6^. NLRP3 inflammasome is a multi-protein complex comprising a sensor NLRP3, an adaptor molecule apoptosis-associated speck-like protein containing a caspase recruitment domain CARD (ASC), and an effector caspase-1^7^. Pyroptosis is involved in many liver diseases, such as hepatocytic injury and inflammation^8^. Therefore, the inhibition of NLRP3 inflammasome-initiated pyroptosis is a new strategy for the preventing and treating LPS-induced liver injury.

Polyunsaturated fatty acids (PUFAs) are important nutrients in the human body involved in physiological and pathological processes^9,10^. Docosahexaenoic acid (DHA) is the most important one of the omega-3 PUFAs^11^. Previous studies demonstrated that DHA inhibited inflammation and had beneficial effects on various inflammatory diseases^11–14^. Arachidonic acid (AA) is a representative type of omega-6 PUFAs and increasing dietary intake of omega-6 fatty acids increases inflammation^15^. However, studies on healthy adults have found that an increased intake of AA did not increase the concentrations of inflammatory cytokines^16^. Therefore, the role of AA in the inflammatory environment is complex and has not yet been properly understood. Furthermore, whether DHA and AA exert effects on pyroptosis in LPS-induced Kupffer cells has not been reported. G protein-coupled receptor 120 (GPR120, also known as FFAR4: free fatty acid receptor 4) is the receptor for PUFAs^17–19^ and has been located in adipocytes^20^, macrophages^18^, and Kupffer cells^21^. DHA regulated anti-inflammatory and insulin-sensitizing effects through GPR120 signaling^17,18,22,23^. Agonist activation causes the redistribution of GPR120 away from the cell surface into internal cellular compartments through endocytosis known as internalization. Previous studies reported that DHA and AA were associated with marked GPR120 internalization^19,24^. However, whether GPR120 mediates DHA and AA action in pyroptosis and how it works have not been reported.

This study was novel in reporting that DHA/AA alleviated Kupffer cells pyroptosis via GPR120 interaction with NLRP3 in LPS-treated Kupffer cells. Thus, the study provided an in-depth resource and framework for understanding the effects of DHA/AA while suggesting approaches for hepatic injury therapy.

## Results

### DHA/AA inhibited NLRP3 inflammasome-mediated pyroptosis in LPS-induced Kupffer cells

**Fig. 1:**
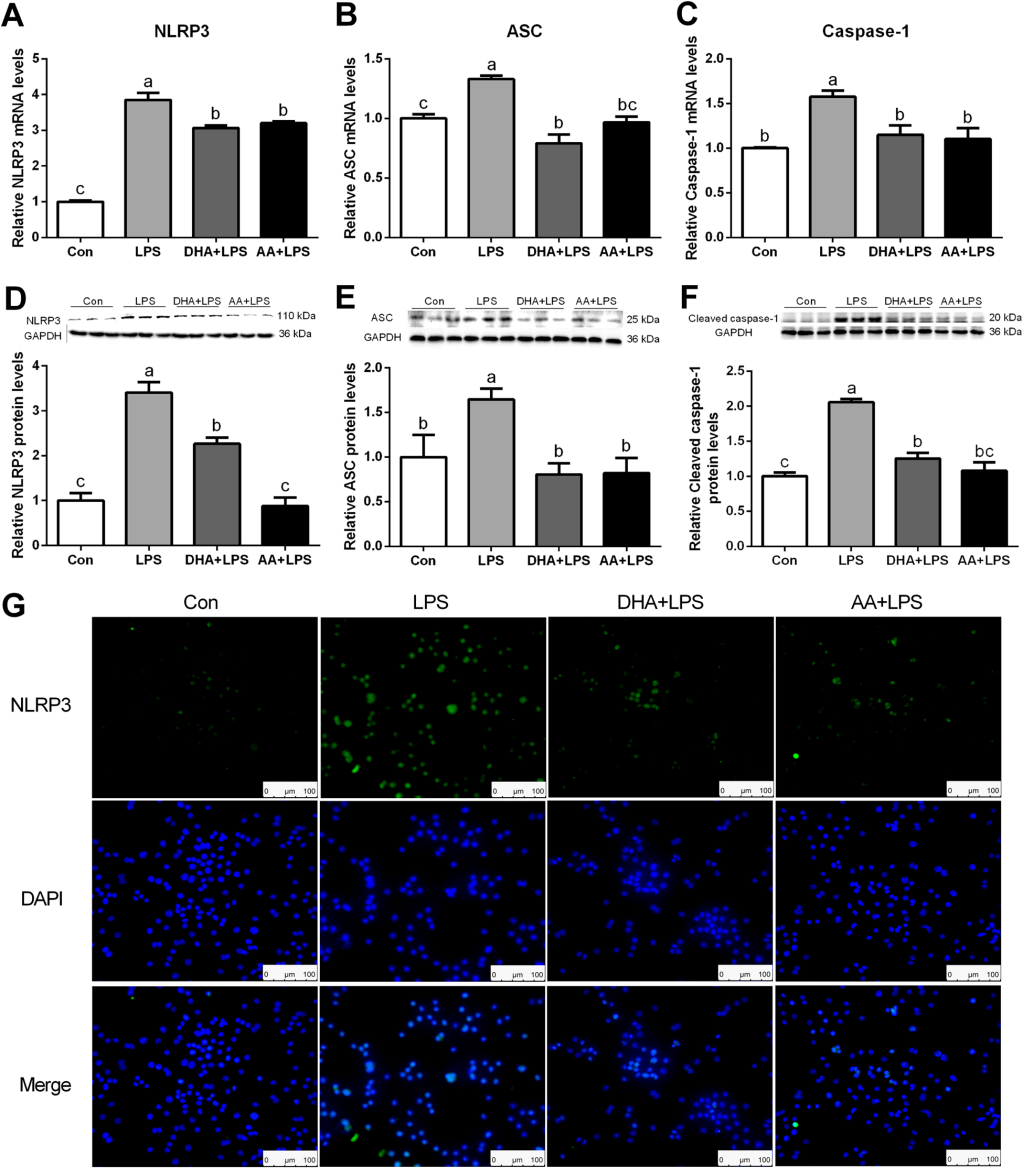
DHA/AA inhibited NLRP3 inflammasome-mediated pyroptosis in LPS-induced Kupffer cells. **A**–**C** Kupffer cells were pretreated with 50 μM DHA/AA for 4 h and then treated with 100 ng/mL LPS for 6 h. The mRNA levels of NLRP3, ASC, and caspase-1 were determined by quantitative real-time PCR. **D**–**F** Kupffer cells were pretreated with 50 μM DHA/AA for 4 h and then were treated with 100 ng/mL LPS for 12 h. The protein levels of NLRP3, ASC, and caspase-1 were determined by western blot analysis. **G** NLRP3 expression was measured by immunofluorescence. In the bar graph, data represent the means ± SEM, *n* = 3 per group. Mean values not sharing the same letters are significantly different, *P* < 0.05.

Various concentrations (0, 10, 50, 100 μM) of DHA/AA treated for 4 h had no effect on cell viability (Supplementary Fig. 1A, B). NLPR3 inflammasome-mediated pyroptosis is a novel programmed cell death that involved in liver inflammation. Pyroptosis was assessed by detecting the level of pyroptosis key proteins to determine the influence of DHA/AA on pyroptosis in LPS-induced Kupffer cells. Quantitative real-time PCR and western blot analysis showed that the expression of NLRP3, ASC, and caspase-1 significantly increased in the LPS group compared with the Con group (Fig. 1A–F). Immunofluorescence analyses further confirmed that the expression of NLRP3 strongly increased in the LPS group compared with the Con group (Fig. 1G). However, the LPS-induced effect was significantly reversed by DHA or AA supplementation.

**Fig. 2:**
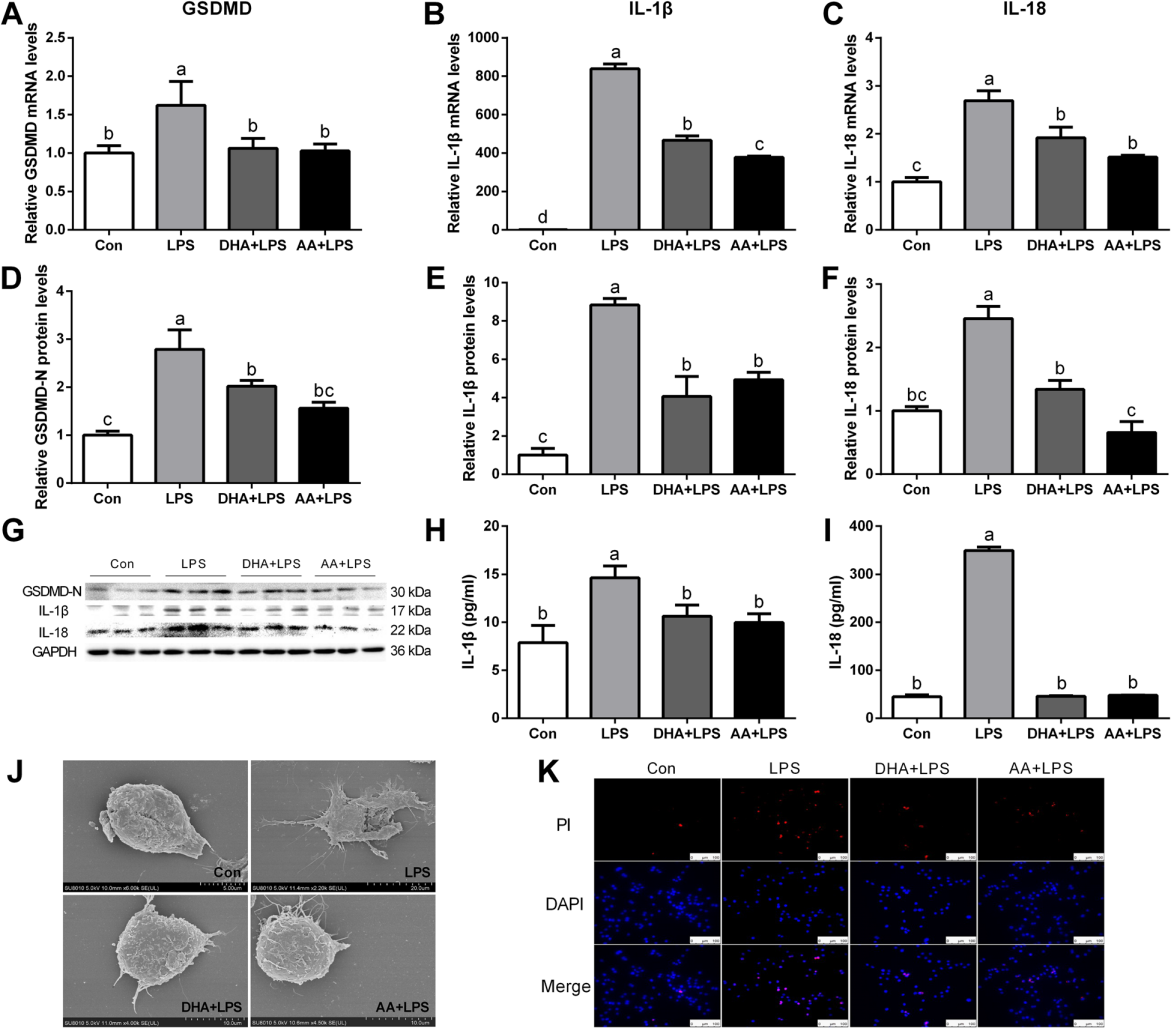
DHA/AA inhibited the expression of GSDMD, IL-1β and IL-18, and cell inflammatory death in LPS-induced Kupffer cells. **A**–**C** Kupffer cells were pretreated with 50 μM DHA/AA for 4 h and then were treated with 100 ng/mL LPS for 6 h. The mRNA levels of GSDMD, IL-1β, and IL-18 were determined by quantitative real-time PCR. **D**–**G** Kupffer cells were pretreated with 50 μM DHA/AA for 4 h and then were treated with 100 ng/mL LPS for 12 h. The protein levels of GSDMD, IL-1β, and IL-18 were determined by western blot analysis. **H**, **I** Levels of IL-1β and IL-18 in the supernatant were measured by ELISA**. J** The cell morphology was observed and imaged under a scanning electron microscope (SEM). **K** PI staining was used in immunofluorescence (scale bar, 100 μm). In the bar graph, data represent the means ± SEM, *n* = 3 per group. Mean values not sharing the same letters are significantly different, *P* < 0.05.

In addition, quantitative real-time PCR and western blot analysis showed that the expression of GSDMD, interleukin-1β (IL-1β), and IL-18 significantly increased in the LPS group compared with the Con group (Fig. 2A–G). Enzyme-linked immunosorbent assay (ELISA) analyses further showed that the secretion of IL-1β and IL-18 significantly increased in the LPS group compared with the Con group (Fig. 2H, I). However, LPS-stimulated expression of GSDMD, IL-1β, and IL-18 was significantly reversed by DHA or AA treatment.

Kupffer cells pyroptosis was detected using scanning electron microscope (SEM). The holes in the cell membrane and the rupture of the cell surface were clearly visible in the LPS group compared with the Con group, while the pyroptosis phenotypes were improved by DHA or AA supplementation (Fig. 2J). Likewise, the result of propidium iodide (PI) staining was consistent with the change in cellular phenotype (Fig. 2K).

### DHA/AA alleviated inflammation response in LPS-induced Kupffer cells

Kupffer cells were treated with DHA/AA at various concentrations and time course to evaluate their effect on inflammatory responses. The expression of IL-1β and IL-18 strongly decreased in the 50 and 100 μΜ DHA/AA group compared with the LPS group (Supplementary Fig. 1C–F). In addition, the expression of IL-1β and IL-18 significantly decreased after treatment with 50 μΜ DHA/AA for 4 or 12 h compared with the LPS group (Supplementary Fig. 1G–J).

Quantitative real-time PCR showed that the mRNA expression of IL-6, MCP-1, and iNOS significantly increased in the LPS treatment group compared with the Con group (Supplementary Fig. 2A–C). Western blot analysis showed that the protein expression of tumor necrosis factor α (TNF-α) and cyclooxygenase-2 (COX2) significantly increased in the LPS group compared with the Con group (Supplementary Fig. 2D–F). However, the expression of IL-6, MCP-1, iNOS, TNF-α, and COX2 significantly decreased in the DHA or AA supplementation group compared with the LPS group (Supplementary Fig. 2A–F).

### Interference with GPR120 eliminated the effects of DHA/AA on NLRP3 inflammasome-mediated Kupffer cells pyroptosis

**Fig. 3:**
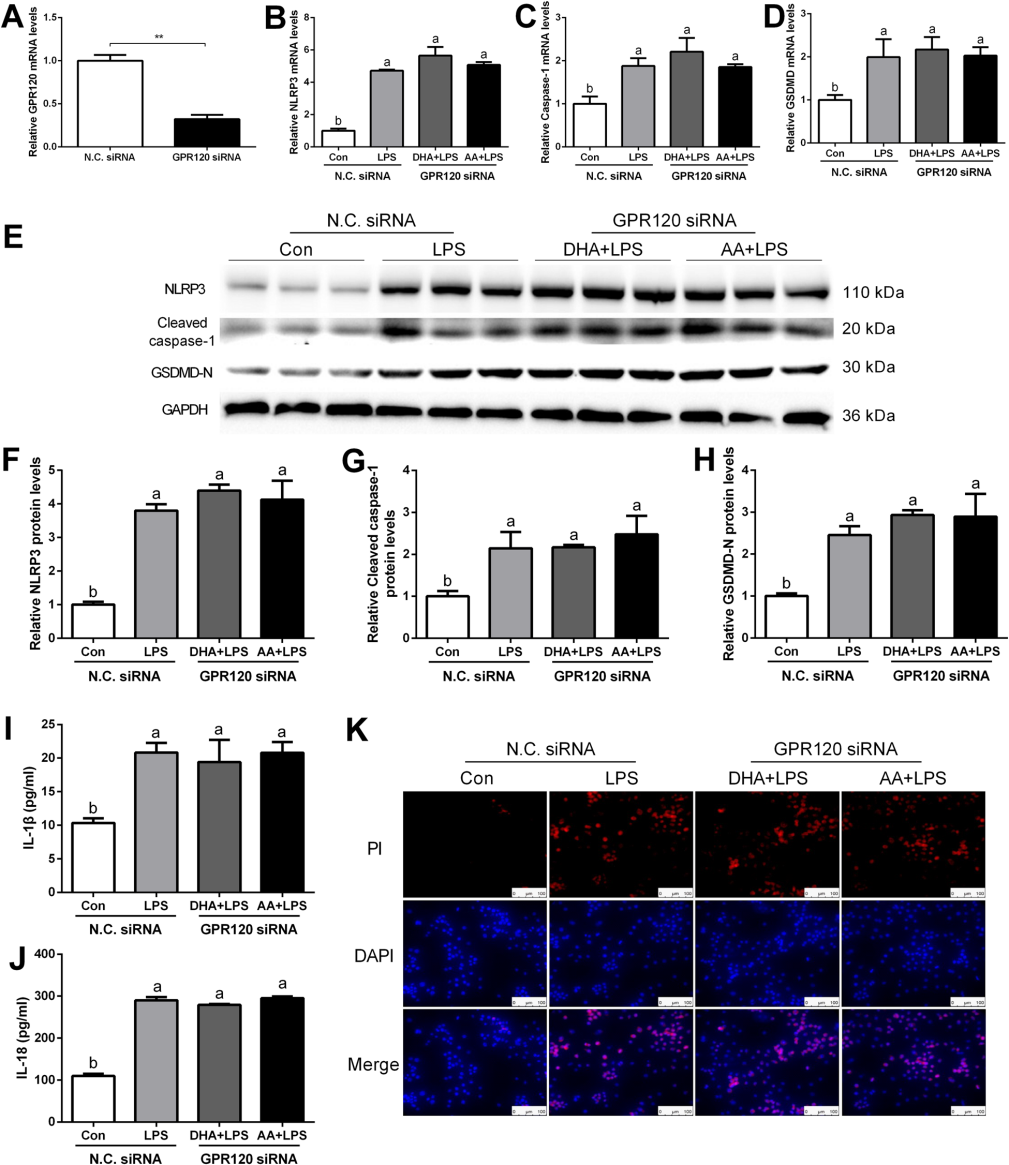
Silencing GPR120 abolished the inhibition of pyroptosis by DHA and AA in LPS-induced Kupffer cells. GPR120 siRNA was transfected into Kupffer cells for 24 h. **A**–**D** mRNA levels of GPR120, NLRP3, caspase-1, and GSDMD were determined by quantitative real-time PCR. **E**–**H** Protein levels of NLRP3, caspase-1, and GSDMD were determined by western blot analysis. **I**, **J** Levels of IL-1β and IL-18 in the supernatant were measured by ELISA; **K** PI staining was used in immunofluorescence (scale bar, 100 μm). In the bar graph, data represent the means ± SEM, *n* = 3 per group. Mean values not sharing the same letters are significantly different, *P* < 0.05; **P < 0.01.

Previous studies demonstrated that DHA anti-inflammatory activity in macrophages was GPR120-dependent^13,18^. However, whether AA can activate GPR120 or whether DHA and AA can exert anti-pyroptosis effects through GPR120 was unclear. To address this, si-GPR120 (siRNA1, siRNA2, and siRNA3) was transfected to achieve GPR120 silencing, and quantitative real-time PCR (Supplementary Fig. 3A) and western blot analysis were performed to verify the transfection efficiency (Supplementary Fig. 3B). Subsequently, GPR120 siRNA3 was chosen for subsequent experiments and the mRNA expression of GPR120 significantly decreased after using GPR120 siRNA compared with the NC siRNA group (Fig. 3A). Quantitative real-time PCR and western blot analysis showed that the expression of NLRP3, caspase-1, and GSDMD significantly increased in the LPS group compared with the Con group (Fig. 3B–H). ELISA analyses further showed that the secretion of IL-1β and IL-18 significantly increased in the LPS group compared with the Con group (Fig. 3I, J). However, the LPS-induced effects were not improved by DHA or AA supplementation after GPR120 silencing. Also, PI staining revealed similar results (Fig. 3K).

### DHA/AA promoted GPR120 interaction with NLRP3 and decreased NLRP3 inflammasome assembly in LPS-induced Kupffer cells

**Fig. 4:**
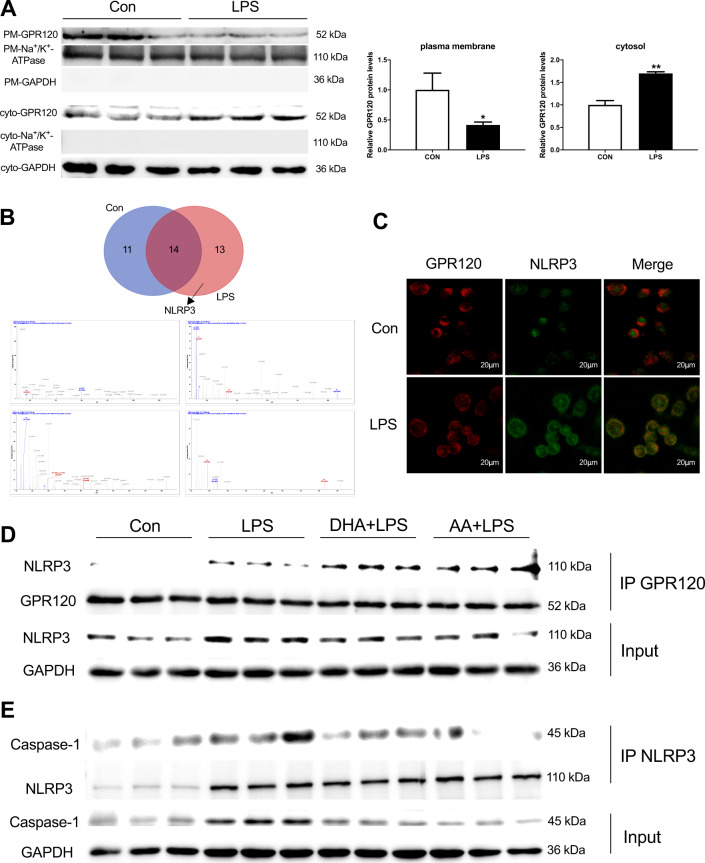
DHA/AA promoted the interaction between GPR120 and NLRP3 in LPS-induced Kupffer cells. Kupffer cells were treated with 100 ng/mL LPS for 12 h. **A** The GPR120 protein expression in the plasma membrane and cytoplasm. **B** The expression of NLRP3 protein was observed by the Venn diagram and LC-MS/MS analysis (the upper image is the Venn diagram and the lower one is the NLRP3 mass spectrogram). **C** GPR120 and NLRP3 co-localization was observed in immunofluorescence (scale bar, 20 μm); **D**, **E** Kupffer cells were pretreated with 50 μM DHA/AA for 4 h and then treated with 100 ng/mL LPS for 12 h. The cell lysates were immunoprecipitated with GPR120 antibody and NLRP3 antibody separately, and then the samples were analyzed by western blot for GPR120, NLRP3, and caspase-1 as indicated in the text. In the bar graph, data represent the means ± SEM, *n* = 3 per group. **P < 0.01 and *P < 0.05.

Kupffer cells were treated with LPS for 12 h to examine how GPR120 affected pyroptosis. The GPR120 protein expression significantly decreased in the plasma membrane after LPS treatment. In contrast, LPS stimulation significantly increased the protein expression of GPR120 in the cytoplasm (Fig. 4A). Then, the samples for the immunoprecipitation (IP) of GPR120 were used to detect the protein that interacted with GPR120 with the help of liquid chromatography with tandem mass spectrometry (LC-MS/MS). Venn diagram showed the detected protein in the Con and LPS groups; NLRP3 was observed in the LPS group (Fig. 4B). In addition, immunofluorescence revealed the co-localization of GPR120 and NLRP3 in the LPS group (Fig. 4C). Next, co-IP was used to explore whether the interaction between GPR120 and NLRP3 contributed to the anti-pyroptosis effects of DHA/AA. The results shown in Fig. 4D indicated that DHA and AA promoted the interaction between GPR120 and NLRP3, thus decreasing the self-assembly of inflammasome complex. In addition, the decreased interaction between NLRP3 and caspase-1 was observed after DHA and AA supplementation (Fig. 4E). This finding further confirmed that DHA and AA via GPR120 interacted with NLRP3 and decreased NLRP3 inflammasome assembly to alleviate LPS-induced Kupffer cells pyroptosis.

### DHA/AA protected mice from LPS-induced hepatic injury

**Fig. 5:**
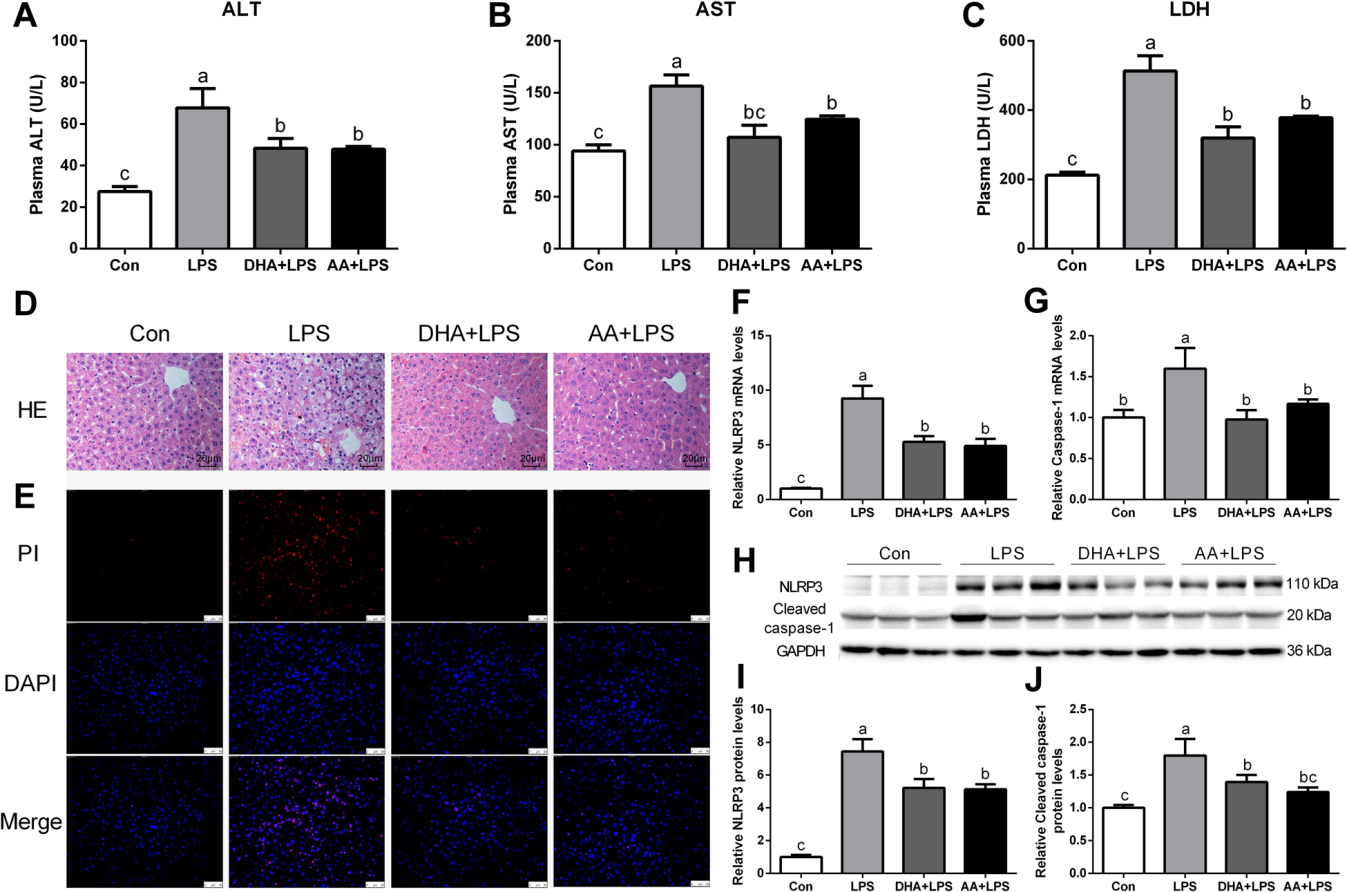
DHA/AA ameliorated hepatic injury in LPS-induced mice. **A**–**C** Plasma ALT, AST, and LDH levels were measured (*n* = 6 mice per group). **D** Representative images of hematoxylin and eosin staining (scale bar, 20 μm, *n* = 3 mice per group). **E** Immunofluorescence images of PI staining (scale bar, 50 μm, *n* = 3 mice per group). **F**, **G** mRNA levels of NLRP3 and caspase-1 were determined by quantitative real-time PCR (*n* = 6 mice per group). **H**–**J** Protein levels of NLRP3 and caspase-1 were determined by western blot analysis (*n* = 6 mice per group). In the bar graph, data represent the means ± SEM. Mean values not sharing the same letters are significantly different, *P* < 0.05.

Pretreatment with DHA and AA significantly improved LPS-induced liver injury as evidenced by reduced plasma alanine aminotransferase (ALT), aspartate transaminase (AST), and lactate dehydrogenase (LDH) levels (Fig. 5A–C). Also, hematoxylin and eosin (H&E) staining showed that inflammatory cell infiltration was clearly visible in the LPS group compared with the Con group, while the hepatic injury phenotypes were improved by DHA or AA supplementation (Fig. 5D). Likewise, the result of PI staining was consistent with the change in the hepatic phenotype (Fig. 5E). Quantitative real-time PCR and western blot analysis showed that the expression of NLRP3 and caspase-1 significantly increased in the LPS group compared with the Con group (Fig. 5F–J). The LPS-induced effects were significantly ameliorated by DHA or AA supplementation.

### DHA/AA alleviated pyroptosis in LPS-induced primary Kupffer cells

**Fig. 6:**
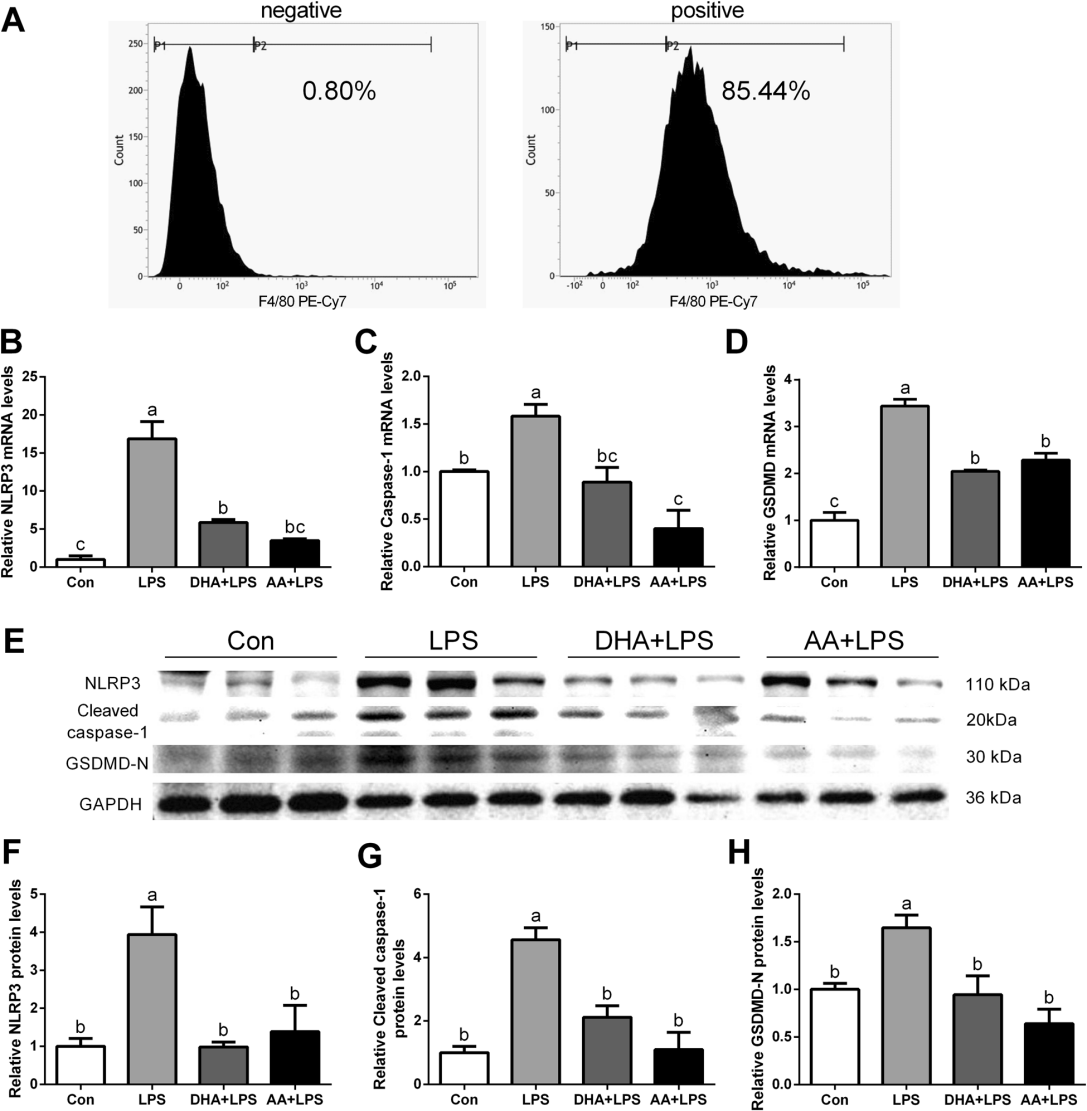
DHA/AA improved pyroptosis in primary Kupffer cells of LPS-induced mice. **A** F4/80-positive cells accounted for 85.44% of total cells in the positive group and 0.80% in the negative group. **B**–**D** mRNA levels of NLRP3, caspase-1, and GSDMD were determined by quantitative real-time PCR. **E–H** Protein levels of NLRP3, caspase-1, and GSDMD were determined by western blot analysis. In the bar graph, data represent the means ± SEM, *n* = 3 mice per group. Mean values not sharing the same letters are significantly different, *P* < 0.05.

Primary Kupffer cells were isolated from the livers of LPS-exposed mice after pretreatment with DHA and AA. The purity of the Kupffer cells fraction was determined using the F4/80 antibody. The results of flow cytometry showed that F4/80-positive cells accounted for 85.44% of all cells (Fig. 6A). Quantitative real-time PCR and western blot analysis showed that the expression of NLRP3, caspase-1, and GSDMD significantly increased in the LPS group compared with the Con group (Fig. 6B–H). The expression of NLRP3, caspase-1, and GSDMD significantly decreased in the DHA or AA supplementation group compared with the LPS group (Fig. 6B–H).

## Discussion

Kupffer cells, as the main macrophages in the liver, have been shown to exert significant effects in some liver diseases^25–27^. This study showed that DHA and AA exhibited anti-pyroptosis activity in LPS-exposed Kupffer cells. The effects of DHA and AA were achieved through the interaction between GPR120 and NLRP3. Moreover, in vivo, DHA and AA supplementation successfully improved LPS-induced hepatic injury in mice by alleviating Kupffer cells pyroptosis.

Pyroptosis has been implicated in various diseases, such as inflammation, nonalcoholic fatty liver disease, and liver fibrosis^28–31^. Bacteria, toxins and so on can elicit a strong immune response in macrophages, resulting in pyroptosis^29,32^. However, the molecular basis of Kupffer cells pyroptosis is incompletely understood, which has limited the identification of therapeutic targets. DHA and AA are both long-chain PUFAs. DHA was found to suppress the progression of hepatic inflammation and inhibits hepatocyte pyroptosis induced by hepatic ischemia–reperfusion^33^. However, the effect of DHA on LPS-induced Kupffer cells pyroptosis and its mechanism are still unclear. In addition, the role of AA in the inflammatory environment is controversial and has not yet been properly understood. Thus, Kupffer cells were pretreated with DHA/AA and then treated with LPS to determine the influence of DHA/AA on pyroptosis in this study. Pyroptosis is inflammatory cell death mediated by NLRP3 inflammasome-dependent activation of caspase-1^34,35^. The ASC participates in the NLRP3 inflammasome mediated activation of caspase-1 and forms activated inflammasome complexes^36^. The activation of caspase-1 leads to the maturation of proinflammatory cytokines IL-1β and IL-18, which are released from the GSDMD pores on the plasma membranes and stimulate the immune cells to induce an inflammatory form of cell death known as pyroptosis^37^. This study was novel in reporting that DHA and AA alleviated the progression of pyroptosis in LPS-exposed Kupffer cells. In addition, pyroptosis is a kind of programmed cell death characterized by cell membrane rupture and perforation. This study showed that the pyroptosis phenotypes were improved by DHA or AA supplementation in LPS-exposed Kupffer cells.

GPR120 recognizes PUFAs^17–19^ and is expressed in Kupffer cells^21^, macrophages^18^, adipose tissue^20^, and intestinal tract^17^. Therefore, GPR120 siRNA was synthesized to transfect Kupffer cells so as to elucidate whether DHA and AA alleviated pyroptosis through GPR120 signaling. The results showed that GPR120 silencing tended to abolish these anti-pyroptosis actions of DHA and AA in LPS-exposed Kupffer cells. These results suggested that DHA and AA alleviated pyroptosis of LPS-induced Kupffer cells via GPR120.

The study further investigated the mechanism underlying the interaction between GPR120 and pyroptosis. Previous studies reported that GPR120 mediated anti-inflammatory effects via internalization^18^. First, the study revealed that a weaker GPR120 plasma membrane signal contrasted with a stronger GPR120 cytoplasmic signal in the LPS group compared with the Con group, suggesting that GPR120 was transferred from the plasma membrane to the cytoplasm to function after LPS treatment. The anti-inflammatory effects mediated by GPR120 depended on its binding to downstream target molecules^38,39^. The study demonstrated GPR120 binding to NLRP3 using LC-MS/MS and immunofluorescence after LPS treatment. Next, co-IP was used to explore whether the interaction between GPR120 and NLRP3 contributed to the anti-pyroptosis effects of DHA and AA. The results showed that DHA and AA promoted the interaction between GPR120 and NLRP3, thus inhibiting the self-assembly of the NLRP3 inflammasome complex. The formation of the NLRP3 inflammasome complex requires caspase-1 involvement^40^. This study found that the supplementation with DHA and AA reduced the binding of between NLRP3 and caspase-1. This finding further confirmed that DHA and AA via GPR120 interacted with NLRP3 and inhibited the NLRP3 inflammasome complex assembly to alleviate LPS-induced Kupffer cells pyroptosis.

In support of these findings, mice were pretreated with DHA and AA in vivo and then intraperitoneally injected with LPS to induce hepatic injury and pyroptosis. Previous studies reported that omega-3 PUFA prevented diet-induced nonalcoholic steatohepatitis^13^.The results showed that DHA and AA significantly improved LPS-induced liver injury. In addition, DHA and AA significantly inhibited the expression of NLRP3 and caspase-1 in LPS-treated mouse liver. Previous studies showed that GSDMD-driven pyroptosis in immune cells aggravated hepatic ischemia–reperfusion injury^41^. Therefore, primary Kupffer cells were isolated from the livers of LPS-exposed mice after pretreatment with DHA and AA. The study showed a significant decrease in primary Kupffer cells pyroptosis in LPS-exposed mice pretreated with DHA and AA, suggesting that DHA and AA might alleviate liver injury though inhibiting Kupffer cells pyroptosis.

Taken together, the data showed that DHA and AA alleviated LPS-induced Kupffer cells pyroptosis via GPR120 interaction with NLRP3 to inhibit NLRP3 inflammasome complex assembly. These findings provided new insights into the treatment of hepatic injury.

## Material and methods

### Regents

DHA (HY-B2167) was purchased from MCE (MedChemExpress, New Jersey, USA) and AA (C4223) were purchased from APExBIO (Achieve Perfection, Explore the Unknown, Houston, USA). LPSs from *Escherichia coli* O55:B5 (LPS, L2880) and 4′,6-diamidino-2-phenylindole (DAPI, D9542) were obtained from Sigma-Aldrich. NLRP3 (BS90949: Bioworld, diluted 1:100) and GPR120 (sc-390752: Santa Cruz, diluted 1:50) were used for immunofluorescence. PI (SL7090) was purchased from Coolaber Science & Technology Co., Ltd (Beijing, China).

### Cell culture and cell transfection

Kupffer cells (NASH-associated mouse Kupffer cell lines, BeNa Culture Collection, China, BNCC340733) were cultured in Roswell Park Memorial Institute 1640 medium (Cat No. 350-000-CL, Wisent, Nanjing, China) containing 10% (v/v) fetal bovine serum at 37 °C in an atmosphere of 5% CO_2_. At 70% confluent growth Kupffer cells were pretreated with 50 μM DHA/AA for 4 h and then treated with 100 ng/mL LPS for 6 or 12 h.

For GPR120 knockdown, specific small interfering RNAs (GPR120 siRNA1, GPR120 siRNA2, and GPR120 siRNA3; GenePharma, Shanghai, China) were transfected into Kupffer cells using jetPRIME^®^ transfection reagent (Polyplus Transfection, Beijing, China). The scramble siRNA was used as a negative control (NC siRNA). The sequences are listed in Supplementary Table S1.

### Animal experiment

A total of 36 male specific-pathogen-free, aged 6 weeks, C57BL/6J mice were purchased from Yangzhou University Comparative Medical Center. All the mice were housed at 22 ± 1 °C, with a 12-h light/dark cycle and fed in the Animal Experiment Center, Nanjing Agricultural University. The mice were allowed to adapt to their environment for 1 week. After that, the mice were randomly assigned to four groups: the control (Con) group (*n* = 9), the LPS group (*n* = 9), DHA and LPS (DHA + LPS) group (*n* = 9), or AA and LPS (AA + LPS) group (*n* = 9). The mice in the DHA + LPS and AA + LPS groups were treated with 50 mg/kg DHA and AA, respectively. DHA and AA were dissolved in 5% gum arabic solution and administered orally once daily via a gastric tube for 1 week before LPS treatment. The mice in the Con and LPS groups were gavaged with an equal volume of 5% gum arabic solution. During the treatment, all mice had free access to water and food. After 1 week of gavage, the mice in the LPS, DHA + LPS, and AA + LPS groups received an intraperitoneal injection of LPS at a dose of 5 mg/kg, and the Con group mice received an intraperitoneal injection of an equal volume of physiological saline solution. After 12 h, the livers from three mice were collected from each group for isolating Kupffer cells, and livers and blood from the other six mice were collected and stored in –80 °C for later use.

This study was approved by the Animal Ethics Committee of Nanjing Agricultural University, China. Euthanasia and sampling procedures complied with the “Guidelines on Ethical Treatment of Experimental Animals” (2006) No. 398 published by the Ministry of Science and Technology, China, and with the “Regulation Regarding the Management and Treatment of Experimental Animals” (2008) No. 45, published by the Jiangsu Provincial People’s Government.

### Primary Kupffer cells isolation and identification

Primary Kupffer cells were isolated separately from C57BL/6 mice in the Con group, LPS group, DHA + LPS group, and AA + LPS group as previously described^42^. Briefly, the liver was perfused with 10 mL of phosphate-buffered saline and then digested with 0.1% type IV collagenase. Following digestion, the liver homogenate was filtered through a 75-μm stainless steel wire mesh to remove undigested tissue. The cell suspension was centrifuged at 50*g* (Eppendorf 5810 R, Germany) for 5 min at 4 °C. The top suspension was separated with 60% Percoll and then centrifuged at 2500*g* for 25 min. The darker layer in the middle comprised Kupffer cells. Kupffer cells were identified by flow cytometry using a monoclonal anti-F4/80 antibody (123114; Biolegend, California, USA) conjugated with PE/Cyanine7 (PE-Cy7). Flow cytometry was performed for detecting the percentage of F4/80-positive cells. Briefly, the collected cells were incubated with the antibody (1:100 dilution) for 30 min at 37 °C. The data were collected using a flow cytometer.

### CCK-8 cell viability assay

Kupffer cells were seeded in a 96-well plate. After 12 h, cells were exposed to different concentrations of DHA/AA for 4 h and then incubated with 10 μL CCK-8 (K1018, APExBIO, Houston, USA)) reagent for 2 h. Absorbance was measured at 450 nm as an indicator of cell activity.

### Immunofluorescence

Kupffer cells were fixed with 4% paraformaldehyde for 10 min. Each section was soaked with in Tris-buffered saline containing 0.3% Triton X-100 for 1 h, blocked with 10% goat serum, and incubated with the primary antibody overnight at 4 °C and then with the secondary antibody. DAPI was used as a marker for cell nuclei.

### SEM observation

Kupffer cells were prepared and fixed with 2.5 % glutaraldehyde, rinsed with phosphate buffer three times, and dehydrated with gradient ethanol of 50%, 70%, 80%, and 90% for 15 min each and then with 100% ethanol three times. Next, the samples were replaced with t-butanol three times, dried with a freeze-drying apparatus, and then glued to the sample stage. Finally, the samples were plated with a 10-nm gold film with a ion-sputtering apparatus, and the cell morphology was observed under an SEM (Hitachi SU8010, Hitachi Technologies, Tokyo, Japan).

### Plasma biochemical measurement

Plasma ALT, AST, and LDH levels were measured by using an automatic biochemical analyzer (7020, Hitachi, Japan).

### H&E staining

Fresh livers were fixed with 4% paraformaldehyde and paraffin-embedded, and the sections were stained using H&E.

### PI staining

Kupffer cells were fixed with 4% paraformaldehyde for 10 min. The liver tissues were fixed with 4% paraformaldehyde for 24 h, paraffin-embedded, and sectioned at a thickness of 5 mm. The tissue was then dewaxed in xylene and antigen repair was performed by boiling the sections in the citric acid buffer for 15 min, cooling for 20 min. And then incubating with 4 μM PI dye for 30 min at 37 °C. DAPI was used as a marker for cell nuclei. The Kupffer cells and liver sections were observed using a fluorescent microscope.

### Isolation and analysis of cellular plasma membrane fractions

Ten centimeter dishes of cells were treated with or without 100 ng/mL LPS for 12 h. Cells were harvested, resuspended in Buffer A (10 mM HEPES, pH 7.4, 1 mM EDTA, 250 mM sucrose) with protease inhibitors, and homogenized with 22G needles for 30 times. The homogenate was then centrifuged at 800*g* for 10 min to remove the nuclear pellet. Membrane fractions were prepared by centrifuging at 20,000*g* for 1 h. The supernatant was collected and designated “cytoplasm extracts”. The precipitation is membrane fractions and were then solubilized in 100 μL Buffer B (150 mM NaCl, 50 mM Tris-Cl pH7.4, 5 mM EGTA, 5 mM EDTA) containing 8 M urea and 2% SDS. The GPR120 levels in plasma membrane fractions were analyzed by western blot.

### Co-immunoprecipitation

Kupffer cells proteins were extracted, and GPR120 and NLRP3 were immunoprecipitated using protein A/G plus agarose (sc-2003, Santa Cruz, CA, USA) as described previously^43^ The expression of GPR120, NLRP3, and caspase-1 was detected using western blot analysis.

### Total RNA isolation and quantitative real-time polymerase chain reaction

Total RNA was isolated from Kupffer cells or liver tissues using TRIzol reagent (Invitrogen, California, USA) treated with RNase-free DNase, and reverse-transcribed to cDNA using random hexamer primers (Promega). Two microliters of diluted cDNA (1:20, *v*/*v*) was used for real-time PCR with an Mx3000P Real-Time Polymerase Chain Reaction (PCR) System (Stratagene). Glyceraldehyde-3-phosphate dehydrogenase (GAPDH) was chosen as a reference gene. All primers were synthesized by Genewiz (Suzhou, China). The primer sequences for qPCR are listed in Supplementary Table S2.

### Total protein extraction and western blot analysis

Total protein was extracted from Kupffer cells or liver tissues as described previously^44^. The protein concentration was determined following the manufacturer’s protocols on the BCA Protein Assay kit (Pierce). For 10% SDS-PAGE, 30 or 50 µg proteins were used. Western blot analysis of target proteins was carried out following the protocols provided by the manufacturer. The antibodies used in western blot analysis are listed in Supplementary Table S3. Images were captured using Tannon-5200 (Shanghai, China) and the band density was analyzed using Image J software. For these specific proteins, GAPDH was used as a loading control.

### Enzyme-linked immunosorbent assay

ELISA kits were used to detect the levels of IL-1β and IL-18 (Multisciences (Lianke) Biotech, Co., Ltd, Hangzhou, China) following the manufacturer’s protocols.

### LC-MS/MS analyses

The protein solution of immunoprecipitated GPR120 was analyzed by LC-MSMS LC-MS/MS following the manufacturer’s protocols.

### Statistical analyses

All statistical analyses were performed using the SPSS 20.0 software for Windows (IBM Corp). All data were expressed as the mean ± SEM. Statistical analyses were performed using either Student’s *t*-test (two-group comparison) or one-way ANOVA analysis of variance (more than two groups) followed by post hoc comparison. For all analyses, the significance level threshold was set at *P* < 0.05.

## Supplementary information

Supplementary Fig. 1

Supplementary Fig. 2

Supplementary Fig. 3

Supplementary Figure legends

Supplementary tables
