# Supplementary figures and images for: DHA/AA alleviates LPS-induced Kupffer cells pyroptosis via GPR120 interaction with NLRP3 to inhibit inflammasome complexes assembly
Source: Cell Death Dis. 2021 Jan 12;12(1):73. doi: 10.1038/s41419-020-03347-3 (PMC7803970; doi:10.1038/s41419-020-03347-3)

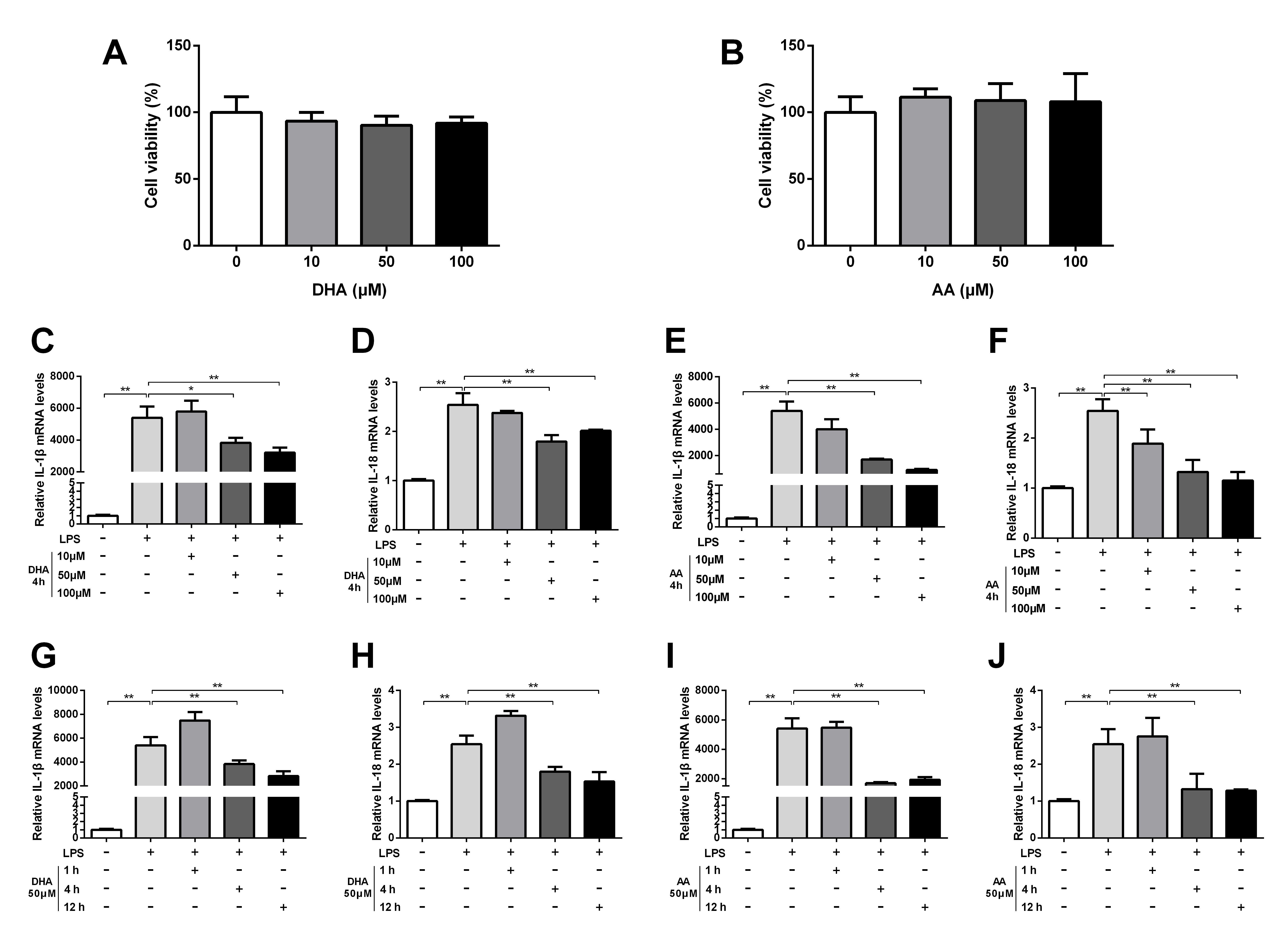

Supplement: Supplementary file 1 — Supplementary Fig. 1 [file 41419_2020_3347_MOESM1_ESM.tif]

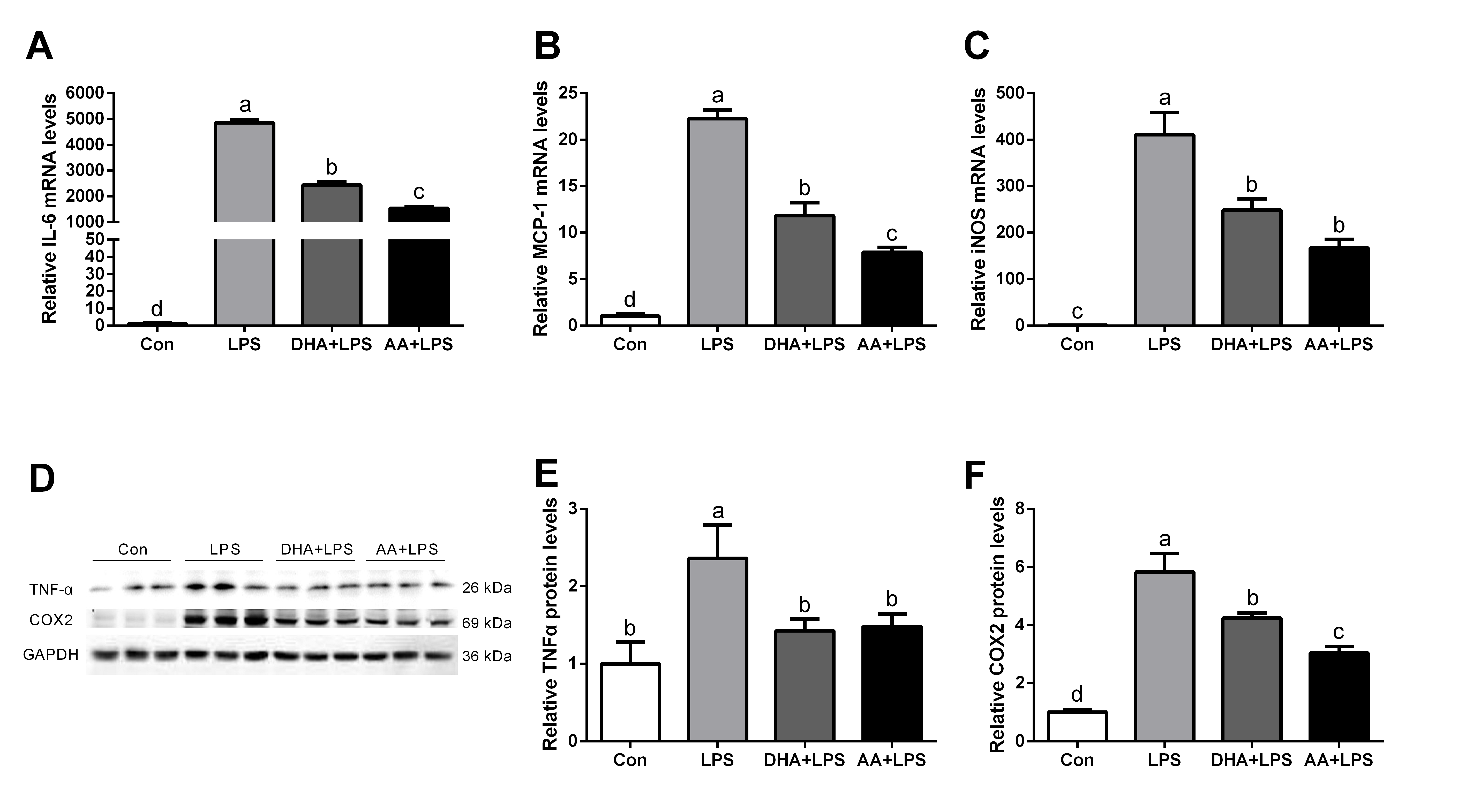

Supplement: Supplementary file 2 — Supplementary Fig. 2 [file 41419_2020_3347_MOESM2_ESM.tif]

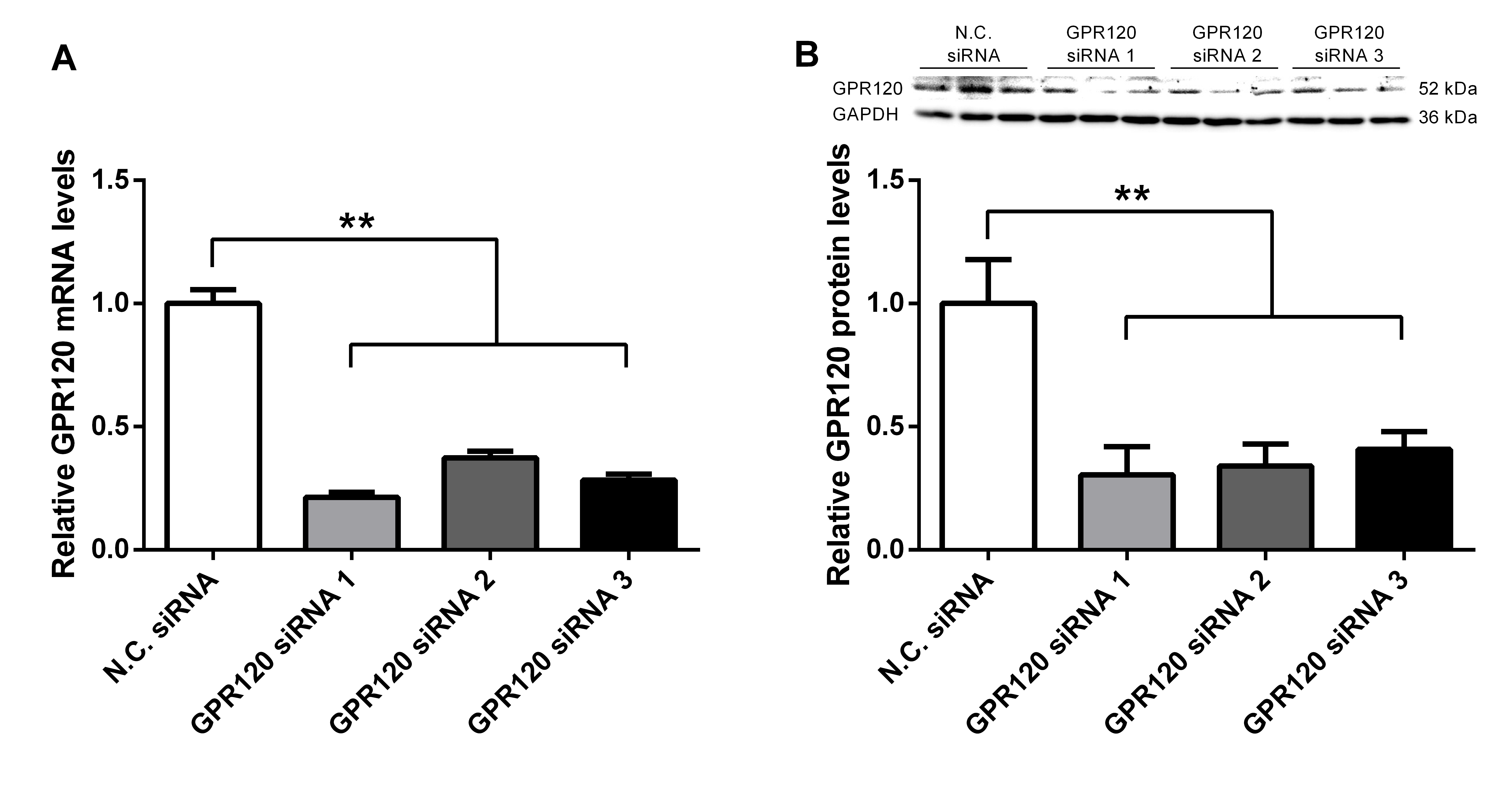

Supplement: Supplementary file 3 — Supplementary Fig. 3 [file 41419_2020_3347_MOESM3_ESM.tif]
