# Supplementary material for: DHA/AA alleviates LPS-induced Kupffer cells pyroptosis via GPR120 interaction with NLRP3 to inhibit inflammasome complexes assembly
Source: Cell Death Dis. 2021 Jan 12;12(1):73. doi: 10.1038/s41419-020-03347-3 (PMC7803970; doi:10.1038/s41419-020-03347-3)
Supplement: Supplementary file 4 — Supplementary Figure legends [file 41419_2020_3347_MOESM4_ESM.docx]

**Supplementary Fig. 1 The effects of different doses and time of DHA/AA on Kupffer cells.** a-b The Kupffer cell viability. c-j The mRNA levels of IL-1β and IL-18 were determined by quantitative real-time PCR. In the bar graph, data represent the means ± SEM, n = 3 per group. **P* < 0.05, ***P* < 0.01.

**Supplementary Fig. 2 DHA/AA alleviated inflammatory responses in LPS-induced Kupffer cells.** a-c Kupffer cells were pretreated with 50 μM DHA/AA for 4 h and then treated with 100 ng/mL LPS for 6 h. The mRNA levels of IL-6, MCP-1, and iNOS were determined by quantitative real-time PCR. d-f Kupffer cells were pretreated with 50 μM DHA/AA for 4 h and then treated with 100 ng/mL LPS for 12 h. Protein levels of TNF-α and COX2 were determined by Western blot analysis. In the bar graph, data represent the means ± SEM, n = 3 per group. Mean values not sharing the same letters are significantly different, *P* < 0.05.

**Supplementary Fig. 3 Interference efficiency of transfected GPR120 siRNA were determined by quantitative real-time PCR and Western blot analysis.** a GPR120 mRNA expression. b GPR120 protein expression. In the bar graph, data represent the means ± SEM, n = 3 per group. ***P* < 0.01 vs the N.C. siRNA group.
