# Supplementary material for: DHA/AA alleviates LPS-induced Kupffer cells pyroptosis via GPR120 interaction with NLRP3 to inhibit inflammasome complexes assembly
Source: Cell Death Dis. 2021 Jan 12;12(1):73. doi: 10.1038/s41419-020-03347-3 (PMC7803970; doi:10.1038/s41419-020-03347-3)
Supplement: Supplementary file 5 — Supplementary tables [file 41419_2020_3347_MOESM5_ESM.doc]

**Table S1: The sequences of GPR120 siRNA**

| siRNA | Sense | Antisense |
| --- | --- | --- |
| GPR120 siRNA 1 | GCUCAUCUUUGUCGUCUCATT | UGAGACGACAAAGAUGAGCTT |
| GPR120 siRNA 2 | GCACAUUGGAUUGGCCCAATT | UUGGGCCAAUCCAAUGUGCTT |
| GPR120 siRNA 3 | UCACGAAAGCAUCGCGGAATT | UUCCGCGAUGCUUUCGUGATT |

**Table S2: The primer sequences of the target genes**

| Gene | Forward | Reverse |
| --- | --- | --- |
| NLRP3 | CCTTTGAGGCATCCAGGACAA | AAGGTTTGAGGCGGCTTTCT |
| ASC | TGCAACTGCGAGAAGGCTAT | GTGAGCTCCAAGCCATACGA |
| Caspase-1 | CGTACACGTCTTGCCCTCAT | AACTTGAGCTCCAACCCTCG |
| GSDMD | GCAGGAGCAGAGTTCTGTGT | TTGGCTTCCCAAAGGCTAGG |
| IL-1β | CTTCAGGCAGGCAGTATC | CAGCAGGTTATCATCATCATC |
| IL-18 | GTTTATTGACAACACGCTTTAC | AGAGGGTCACAGCCAGTCC |
| IL-6 | ATGGCATCCAAGGAGTGA | GGGAGACAGAAGGGAACAG |
| MCP-1 | CACAACCACCTCAAGCAC | AAGGGAATACCATAACATCA |
| INOS | ACGCTTCACTTCCAATGCAAC | CAGCCTCATGGTAAACACGTTC |
| GPR120 | GCCACCACCCAGAAGAAAA | CCCAACAAGACTACCGACTC |
| GAPDH | TCTCCTGCGACTTCAACA | TGTAGCCGTATTCATTGTCA |

**Table S3: The message of antibodies for western blot**

| **Antibody** | **Company** | **Item No.** | **Dilution ratio** |
| --- | --- | --- | --- |
| NLRP3 | Cell Signalling Technology | 15101 | 1:1000 |
| ASC | Affinity Biosciences | DF6304 | 1:1000 |
| Caspase-1 | Abcam | ab179515 | 1:1000 |
| GSDMD | Abcam | ab219800 | 1:1000 |
| IL-1β | Cell Signalling Technology | 12703S | 1:1000 |
| IL-18 | Bioworld | MB9288 | 1:1000 |
| GPR120 | Bioworld | sc390752 | 1:200 |
| Na+/K+-ATPase | Bioworld | BS90909 | 1:1000 |
| TNF-α | Bioworld | BS5965 | 1:1000 |
| COX2 | Abcam | ab15191 | 1:1000 |
| GAPDH | Bioworld | MB001H | 1:10000 |
